# Fucoidan Attenuates Lead-Induced Liver Injury Associated with IGFBP1 and Gut Microbiota-Derived Tryptophol Metabolism

**DOI:** 10.3390/md24070232

**Published:** 2026-07-02

**Authors:** Dianzun Liu, Kaiyu Shen, Jiaxin Li, Jinrui Miao, Jie Fu, Xianli Liu

**Affiliations:** College of Life Sciences and Medicine, Zhejiang Sci-Tech University, Hangzhou 310018, China; ldzalxy@163.com (D.L.); sky1205@alu.zcmu.edu.cn (K.S.); lijiaxin070115@163.com (J.L.); 15030350627@163.com (J.M.)

**Keywords:** fucoidan, lead-induced liver injury, oxidative stress, IGFBP1, gut microbiota–tryptophan metabolism

## Abstract

Lead (Pb) exposure induces liver injury through oxidative stress, inflammation, and gut–liver axis disruption. This study evaluated the protective effects and associated mechanisms of fucoidan (FU) against Pb-induced liver injury in mice. C57BL/6 mice were exposed to lead acetate and treated with FU. High-dose FU (FU-H) improved food intake, body weight, and liver index; decreased Pb levels in serum and liver; and increased fecal Pb content. Compared with the Model group, FU-H reduced serum ALT, AST, and ALP by 54.8%, 38.6%, and 21.7%, respectively. FU-H restored hepatic SOD and GSH by 10.9% and 46.5% and decreased hepatic MDA by 45.9%; it also restored serum SOD and GSH by 30.4% and 24.0%, decreased serum MDA by 19.6%, and suppressed TNF-α, IL-6, and IL-1β by 15.7%, 21.1%, and 14.9%, respectively. Integrated RNA sequencing and network toxicology suggested that insulin-like growth factor-binding protein 1 (IGFBP1) may be associated with FU-mediated protection, and recombinant IGFBP1 partly weakened FU-associated hepatoprotection. Moreover, 16S rRNA sequencing and untargeted metabolomics showed that FU reshaped Pb-disrupted gut microbiota and altered fecal tryptophan metabolism. Exogenous tryptophol supplementation partially alleviated Pb-induced liver injury. Overall, FU protection was associated with reduced Pb burden, IGFBP1-related redox modulation, and gut microbiota-derived tryptophol metabolism.

## 1. Introduction

Lead (Pb) is a widespread environmental heavy metal pollutant and remains an important global public health concern because of its non-biodegradability, environmental persistence, and bioaccumulative properties [1]. Anthropogenic activities, including mining, smelting, battery manufacturing, and the historical extensive use of leaded gasoline and lead-based paints, can cause long-term contamination of soil, water, and air [2,3]. The general population is mainly exposed to Pb through the ingestion of contaminated food and drinking water, as well as the inhalation of Pb-containing dust or polluted air; in some cases, dermal contact with Pb-containing consumer products may also contribute to Pb exposure [4,5]. Once absorbed, Pb can accumulate in multiple tissues and interfere with normal physiological and biochemical processes, thereby exerting toxic effects on various organs and systems, including the intestine, nervous system, hematopoietic system, reproductive system, kidneys, and liver [6,7,8,9]. Currently, chelation therapy with agents such as calcium disodium ethylenediaminetetraacetic acid (CaNa_2_EDTA) and dimercaptosuccinic acid (DMSA) is the main clinical treatment for Pb poisoning. However, existing chelation therapies still have several limitations, including the potential loss of essential trace elements, risk of nephrotoxicity, insufficient safety for long-term use, and limited efficacy in ameliorating established target-organ damage [10]. Therefore, developing safer, better-tolerated, and more effective pharmacological or adjunctive intervention strategies is of great importance for preventing and treating systemic Pb toxicity and related target-organ injury.

Among the multiple organs affected by Pb exposure, the liver is an important site of Pb accumulation, accounting for approximately 33% of the total absorbed Pb, and is also one of the key target organs of Pb toxicity [11,12]. Previous studies have shown that Pb exposure can induce excessive generation of reactive oxygen species (ROS) and weaken endogenous antioxidant defenses, thereby disrupting hepatic redox homeostasis. Persistent oxidative stress can further trigger lipid peroxidation, protein carbonylation, and DNA damage, ultimately leading to histopathological alterations such as hepatocyte apoptosis, necrosis, and inflammatory cell infiltration [13,14]. Therefore, identifying stress-response molecules associated with hepatic redox homeostasis may help further clarify the molecular basis of Pb-induced liver injury and potential intervention strategies.

Insulin-like growth factor-binding protein 1 (IGFBP1) is a liver-enriched IGF-binding protein that regulates the bioavailability of insulin-like growth factor 1 (IGF-1) and has also been implicated in hepatic metabolic stress, cell survival, and injury repair [15]. Previous studies have reported abnormal IGFBP1 expression in liver diseases closely associated with oxidative stress, including alcoholic liver disease and non-alcoholic fatty liver disease (NAFLD) [16,17]. In chemical liver injury models, changes in IGFBP1 have also been associated with lipid peroxidation, malondialdehyde (MDA) levels, and the extent of hepatocellular injury [18,19]. Given that Pb-induced liver injury is characterized by oxidative stress and inflammatory responses, IGFBP1 may represent a potentially relevant molecular target worthy of further investigation in the context of Pb-induced hepatotoxicity.

Notably, in addition to these direct hepatotoxic effects, the gut microbiota has also been implicated in the development and alleviation of Pb-induced liver injury. Pb exposure can disrupt gut microbial homeostasis, as characterized by reduced microbial diversity, decreased abundance of beneficial bacteria, and an increased risk associated with opportunistic pathogens [20,21]. Conversely, specific probiotics and their metabolites can effectively alleviate liver injury by modulating the gut–liver axis [22]. These findings suggest that regulating gut microbiota and related metabolites may represent an important strategy for intervening in Pb-induced hepatotoxicity.

Fucoidan (FU) is a class of natural dietary polysaccharides mainly derived from brown algae and possesses multiple biological activities, including antioxidant, anti-inflammatory, and gut microbiota-modulating effects [23]. The sulfate groups and fucose residues in its structure are believed to be closely associated with its free radical-scavenging and oxidative stress-regulating capacities, allowing fucoidan to alleviate oxidative damage through electron- or hydrogen-donating mechanisms [24,25]. In recent years, the role of fucoidan in modulating the gut–liver axis has attracted increasing attention. Studies have shown that fucoidan can increase the abundance of beneficial bacteria such as *Lachnospiraceae* [26] and regulate bile acid metabolism and its related farnesoid X receptor (FXR) and Takeda G protein-coupled receptor 5 (TGR5) signaling pathways, thereby improving hepatic metabolic function [27]. Although several natural polysaccharides have been reported to reduce heavy-metal burden and alleviate tissue injury through metal-binding capacity, antioxidant defense, intestinal barrier protection, and gut microbiota regulation [28,29,30,31], direct evidence regarding fucoidan intervention in heavy metal-induced organ injury, particularly Pb-induced liver injury, remains limited. Moreover, previous studies have mainly focused on the overall detoxification effects or single protective pathways of polysaccharides, whereas whether fucoidan can alleviate Pb-induced liver injury through the above multi-target processes remains unclear.

Therefore, in the present study, we established a mouse model of Pb-induced liver injury to evaluate the hepatoprotective effects of fucoidan and to explore its associated molecular and gut–liver axis-related mechanisms. RNA sequencing and network toxicology were used to screen candidate hepatic targets, followed by IGFBP1 intervention experiments. Meanwhile, 16S ribosomal RNA (16S rRNA) sequencing and untargeted metabolomics, combined with tryptophol validation, were performed to investigate gut microbiota remodeling and fecal tryptophan metabolism. This study provides experimental evidence that fucoidan alleviates Pb-induced liver injury and suggests that its protective effects are associated with oxidative stress-related molecular responses and gut–liver axis modulation.

## 2. Results

### 2.1. FU Improves General Conditions and Alters Pb Burden in Pb-Intoxicated Mice

**Figure 1:**
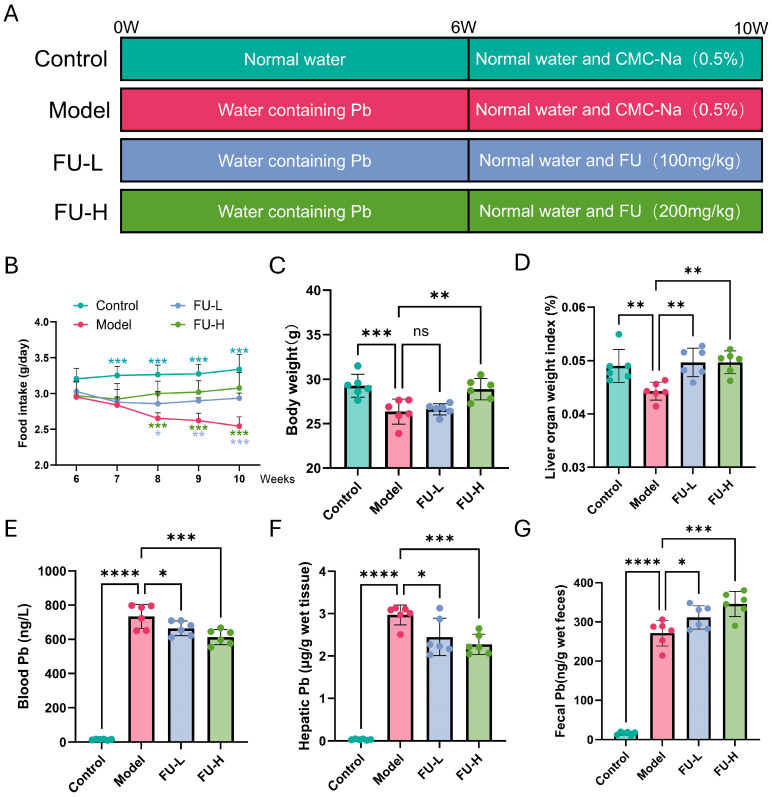
FU improves general conditions and alters Pb burden in Pb-intoxicated mice. (**A**) Schematic illustration of the experimental design. Mice in the Model, FU-L, and FU-H groups were exposed to Pb-containing drinking water for 6 weeks, followed by 4 weeks of intervention with vehicle or FU at 100 mg/kg or 200 mg/kg. The Control group received normal drinking water throughout the experiment; (**B**) weekly food intake of mice from week 6 to week 10; (**C**) body weight of mice at the end of the experiment; (**D**) liver organ weight index; (**E**) blood Pb concentration; (**F**) hepatic Pb content; (**G**) fecal Pb content. Data are presented as mean ± SD, *n* = 6 per group. Statistical significance was analyzed by one-way ANOVA or two-way ANOVA. Compared with the Model group: ns, not significant; * *p* < 0.05, ** *p* < 0.01, *** *p* < 0.001, and **** *p* < 0.0001.

To assess the effect of FU intervention on the general condition of Pb-exposed mice, food intake was monitored during the experimental period, and body weight as well as liver weight were measured at the end of the experiment. As shown in Figure 1A–D, 6 weeks of Pb exposure resulted in reduced food intake, decreased body weight, and a lower liver index, indicating evident toxicity. In contrast, subsequent FU administration after Pb exposure partially restored these changes, suggesting an improvement in the general condition of Pb-exposed mice. Furthermore, chronic Pb exposure also increased lead levels in serum, liver, and feces, reflecting systemic lead accumulation. After 4 weeks of FU oral gavage, Pb content was significantly decreased in both blood and liver, while fecal Pb content was further increased (Figure 1E–G). Importantly, although both FU-L (100 mg/kg) and FU-H (200 mg/kg) exerted significant protective effects, FU-H showed superior improvement in all the above parameters compared to FU-L.

### 2.2. FU Alleviates Pb-Induced Liver Pathological Damage, Oxidative Stress, and Systemic Inflammation

**Figure 2:**
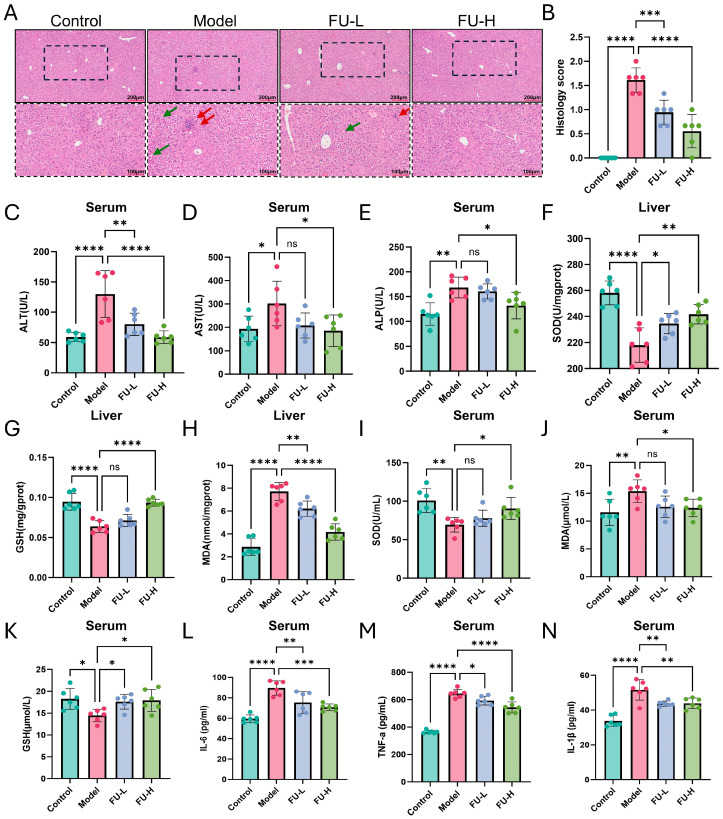
FU alleviates Pb-induced hepatic pathological damage, oxidative stress, and inflammation. (**A**) Representative H&E-stained liver sections from the Control, Model, FU-L, and FU-H groups. The upper panels show 100×-magnification images (scale bar = 200 μm), and the lower panels show enlarged views of the dashed areas (scale bar = 100 μm). Red arrows indicate inflammatory cell infiltration, and green arrows indicate fatty vacuolation; (**B**) histology scores of liver sections; (**C**–**E**) serum levels of liver injury biomarkers, including ALT (**C**), AST (**D**), and ALP (**E**); (**F**–**H**) hepatic oxidative stress-related indicators, including SOD activity (**F**), GSH level (**G**), and MDA level (**H**); (**I**–**K**) serum oxidative stress-related indicators, including SOD activity (**I**), MDA level (**J**), and GSH level (**K**); (**L**–**N**) serum levels of inflammatory cytokines, including IL-6 (**L**), TNF-α (**M**), and IL-1β (**N**). Data are presented as mean ± SD, *n* = 6 per group. Statistical significance was determined by one-way ANOVA. Compared with the Model group: ns, not significant; * *p* < 0.05, ** *p* < 0.01, *** *p* < 0.001, and **** *p* < 0.0001.

Hematoxylin and eosin (H&E) staining showed that Pb exposure induced disordered hepatocyte arrangement, inflammatory cell infiltration, and extensive fatty vacuolation, whereas FU treatment alleviated these pathological alterations (Figure 2A,B). Consistently, Pb exposure markedly increased serum alanine aminotransferase (ALT), aspartate aminotransferase (AST), and alkaline phosphatase (ALP) levels, while FU treatment reduced these indicators. Compared with the Model group, FU-L decreased ALT, AST, and ALP by 38.4%, 31.0%, and 4.7%, respectively, whereas FU-H decreased them by 54.8%, 38.6%, and 21.7%, respectively (Figure 2C–E). For oxidative stress-related indicators, FU-L restored hepatic superoxide dismutase (SOD) and glutathione (GSH) by 7.6% and 11.7%, respectively, and decreased hepatic malondialdehyde (MDA) by 19.5%, whereas FU-H restored hepatic SOD and GSH by 10.9% and 46.5%, respectively, and decreased hepatic MDA by 45.9% (Figure 2F–H). Similar changes were observed in serum, where FU-L restored SOD and GSH by 12.2% and 21.8%, respectively, and decreased MDA by 18.3%, while FU-H restored SOD and GSH by 30.4% and 24.0%, respectively, and decreased MDA by 19.6% (Figure 2I–K). In addition, FU-L reduced serum tumor necrosis factor-α (TNF-α), interleukin-1β (IL-1β), and interleukin-6 (IL-6) by 8.1%, 15.2%, and 15.9%, respectively, whereas FU-H reduced them by 15.7%, 14.9%, and 21.1%, respectively (Figure 2L–N). These results indicate that FU alleviated Pb-induced liver pathological damage, oxidative stress dysregulation, and systemic inflammatory responses.

### 2.3. Integration of Network Toxicology and Transcriptome Sequencing Identifies Molecular Targets of FU Against Pb-Induced Liver Injury

**Figure 3:**
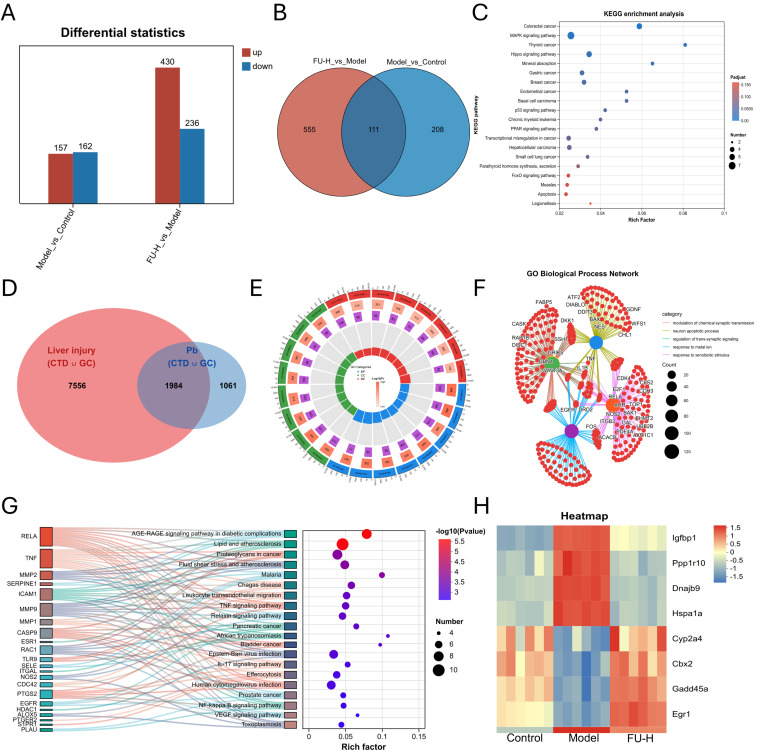
Integrated transcriptomic and network toxicology analyses identify potential molecular targets of FU against Pb-induced liver injury. (**A**) MA plots showing differentially expressed genes in the comparisons of Model versus Control and FU-H versus Model; (**B**) Venn diagram showing the overlap of differentially expressed genes between the Model versus Control and FU-H versus Model comparisons. A total of 111 common differentially expressed genes were identified; (**C**) KEGG pathway enrichment analysis of the overlapping differentially expressed genes. Bubble size represents the number of enriched genes, and bubble color represents the adjusted *p* value; (**D**) Venn diagram showing the intersection between Pb-related targets and liver injury-associated targets identified by network toxicology analysis; (**E**) GO enrichment circular plot of the intersecting targets, including biological process, cellular component, and molecular function categories; (**F**) GO Biological Process network illustrating the relationships between enriched biological processes and their associated genes; (**G**) KEGG pathway enrichment analysis of the intersecting targets obtained from network toxicology analysis; (**H**) heatmap showing the expression profiles of MAPK signaling pathway-related differentially expressed genes among the Control, Model, and FU-H groups. IGFBP1 was identified as the most prominently altered candidate gene associated with FU-mediated protection. Differentially expressed genes were screened using the criteria of |log_2_ fold change| ≥ 1 and adjusted *p* < 0.05. Enrichment analyses were performed based on significantly enriched GO terms and KEGG pathways.

To explore the molecular mechanisms by which FU ameliorates Pb-induced liver injury, we first performed transcriptome sequencing on liver tissues from mice after FU intervention. As shown in Figure 3A, distinct differences in gene expression profiles were observed among the 3 groups. Specifically, compared with the Control group, 319 differentially expressed genes (DEGs) were identified in the Model group (157 up-regulated and 162 down-regulated). Meanwhile, 666 DEGs (430 up-regulated and 236 down-regulated) were found in the FU-H group compared with the Model group. Of note, 111 overlapping DEGs were shared between these two comparisons (Figure 3B). Kyoto Encyclopedia of Genes and Genomes (KEGG) enrichment analysis revealed that these common DEGs were mainly enriched in pathways closely related to mitogen-activated protein kinase (MAPK) signaling, Hippo signaling, p53 signaling, PPAR signaling, and apoptosis (Figure 3C).

Subsequently, a network toxicology approach was employed to identify Pb-associated liver injury targets. Both Pb-related targets and liver injury-related targets were collected from the Comparative Toxicogenomics Database (CTD) and GeneCards (GC) databases. A total of 3045 Pb-related targets and 9584 liver injury-related targets were screened. Venn diagram analysis showed that 1984 overlapping targets were identified (Figure 3D). Gene Ontology (GO) enrichment analysis indicated that these intersecting genes were primarily involved in the regulation of trans-synaptic signaling, modulation of chemical synaptic transmission, and the neuron apoptotic process. Furthermore, a GO Biological Process network was constructed to illustrate the key gene interactions (Figure 3E,F). KEGG pathway analysis suggested that the overlapping targets were mainly enriched in the PI3K-Akt and MAPK signaling pathways, indicating that these pathways may be associated with Pb-induced hepatotoxicity (Figure 3G). Importantly, both the transcriptome sequencing and network toxicology analyses converged on the MAPK signaling pathway. Therefore, we further examined the expression changes of DEGs within the MAPK pathway. Among them, IGFBP1 was identified as the most significantly altered gene (Figure 3H).

**Figure 4:**
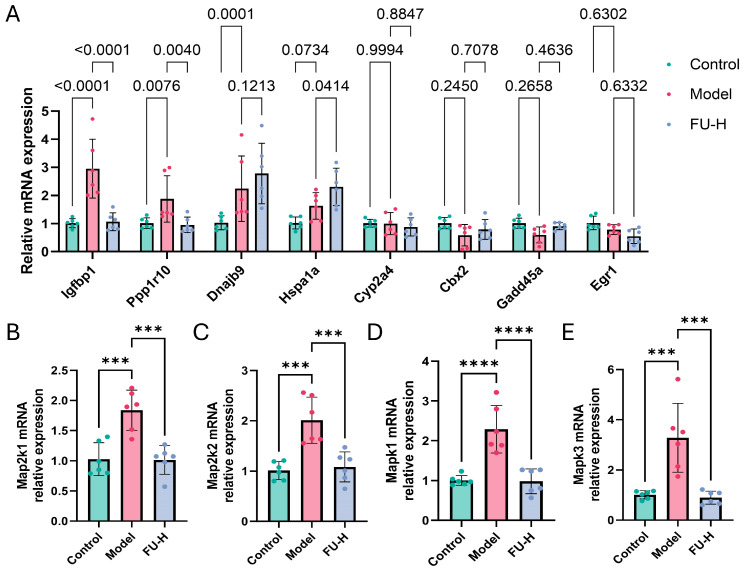
Experimental validation of potential molecular targets involved in FU-mediated protection against Pb-induced liver injury. (**A**) RT-qPCR validation of representative intersecting genes identified by integrated transcriptomic and network toxicology analyses, including *Igfbp1*, *Ppp1r10*, *Dnajb9*, *Hspa1a*, *Cyp2a4*, *Cbx2*, *Gadd45a*, and *Egr1*, in the Control, Model, and FU-H groups; (**B**–**E**) RT-qPCR analysis of MAPK signaling pathway-related genes, including *Map2k1* (**B**), *Map2k2* (**C**), *Mapk1* (**D**), and *Mapk3* (**E**). Data are presented as mean ± SD, *n* = 6 per group. Statistical significance was determined by one-way ANOVA. Compared with the Model group: *** *p* < 0.001, and **** *p* < 0.0001.

To further validate the transcriptomic and network toxicology results, reverse transcription quantitative PCR (RT-qPCR) was performed to examine the expression changes of representative candidate genes. Among these genes, *Igfbp1* exhibited the most pronounced alteration in liver tissues after FU-H intervention (Figure 4A). Previous studies have indicated that the MAPK/extracellular signal-regulated kinase (ERK) signaling pathway can positively regulate the transcriptional expression of IGFBP1 [32]. Therefore, we further examined the mRNA expression levels of core MAPK/ERK pathway-related genes, including *Map2k1*, *Map2k2*, *Mapk1*, and *Mapk3*. The results showed that Pb exposure increased the expression levels of these genes, whereas FU-H intervention partially reversed these changes (Figure 4B–E).

### 2.4. IGFBP1 Is Associated with FU-Mediated Protection Against Pb-Induced Liver Injury

**Figure 5:**
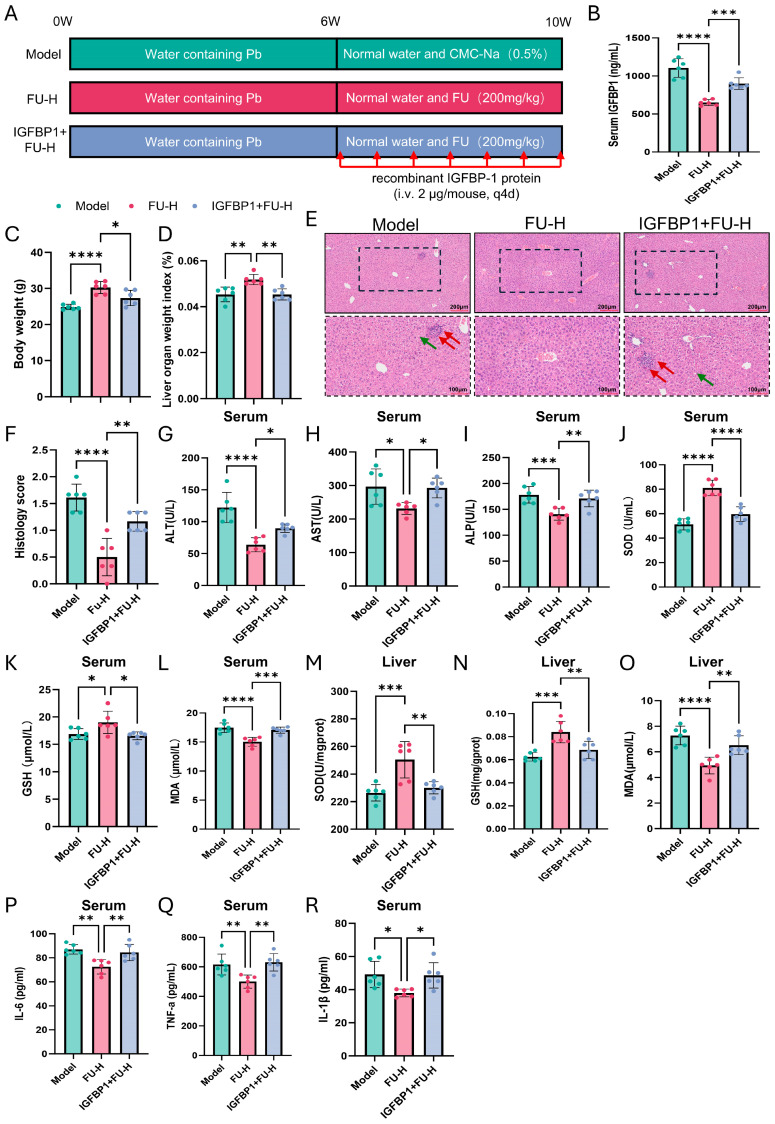
IGFBP1 is associated with FU-mediated protection against Pb-induced liver injury. (**A**) Schematic illustration of the experimental design for IGFBP1 rescue experiments; (**B**) serum IGFBP1 levels in the Model, FU-H, and IGFBP1+FU-H groups; (**C**) body weight of mice at the end of the experiment; (**D**) liver organ weight index; (**E**) representative H&E-stained liver sections from the Model, FU-H, and IGFBP1+FU-H groups. The upper panels show 100×-magnification images (scale bar = 200 μm), and the lower panels show enlarged views of the dashed areas (scale bar = 100 μm). Red arrows indicate inflammatory cell infiltration, and green arrows indicate fatty vacuolation; (**F**) Histology scores of liver sections. (**G**–**I**) Serum levels of hepatic injury biomarkers, including ALT (**G**), AST (**H**), and ALP (**I**); (**J**–**L**) serum oxidative stress-related indicators, including SOD activity (**J**), GSH level (**K**), and MDA level (**L**); (**M**–**O**) hepatic oxidative stress-related indicators, including SOD activity (**M**), GSH level (**N**), and MDA level (**O**). (**P**–**R**) Serum levels of pro-inflammatory cytokines, including IL-6 (**P**), TNF-α (**Q**), and IL-1β (**R**); Data are presented as mean ± SD, *n* = 6 per group. Statistical significance was determined by one-way ANOVA. Compared with the FU-H group: * *p* < 0.05, ** *p* < 0.01, *** *p* < 0.001, and **** *p* < 0.0001.

Based on the transcriptomic, network toxicology, and RT-qPCR validation results, we further explored whether IGFBP1 was functionally associated with FU-mediated protection against Pb-induced liver injury (Figure 5A). Administration of recombinant IGFBP1 increased circulating IGFBP1 levels in mice (Figure 5B). Compared with the FU-H group, co-administration of recombinant IGFBP1 partially weakened the beneficial effects of FU-H on body weight loss and liver index in Pb-exposed mice (Figure 5C,D). Consistently, H&E staining showed that recombinant IGFBP1 partly attenuated the alleviative effect of FU-H on Pb-induced hepatic histopathological alterations (Figure 5E,F).

Furthermore, compared with the FU-H group, the IGFBP1 + FU-H group showed higher serum levels of liver injury markers, with ALT, AST, and ALP increased by 40.9%, 26.4%, and 21.5%, respectively. For oxidative stress-related indicators, IGFBP1 co-administration decreased serum SOD and GSH by 26.5% and 12.9%, respectively, and increased serum MDA by 13.4%. Similarly, hepatic SOD and GSH were decreased by 8.1% and 18.5%, respectively, while hepatic MDA was increased by 32.2% in the IGFBP1 + FU-H group relative to the FU-H group. In addition, the IGFBP1 + FU-H group exhibited increased serum levels of pro-inflammatory cytokines, with IL-6, TNF-α, and IL-1β increased by 16.4%, 26.1%, and 27.8%, respectively, compared with the FU-H group (Figure 5G–R).

Together, these results indicate that exogenous IGFBP1 supplementation partially weakened the protective effects of FU-H in Pb-exposed mice, supporting the involvement of IGFBP1 in FU-associated hepatoprotection.

### 2.5. FU Modulates Pb-Induced Gut Microbiota Dysbiosis

**Figure 6:**
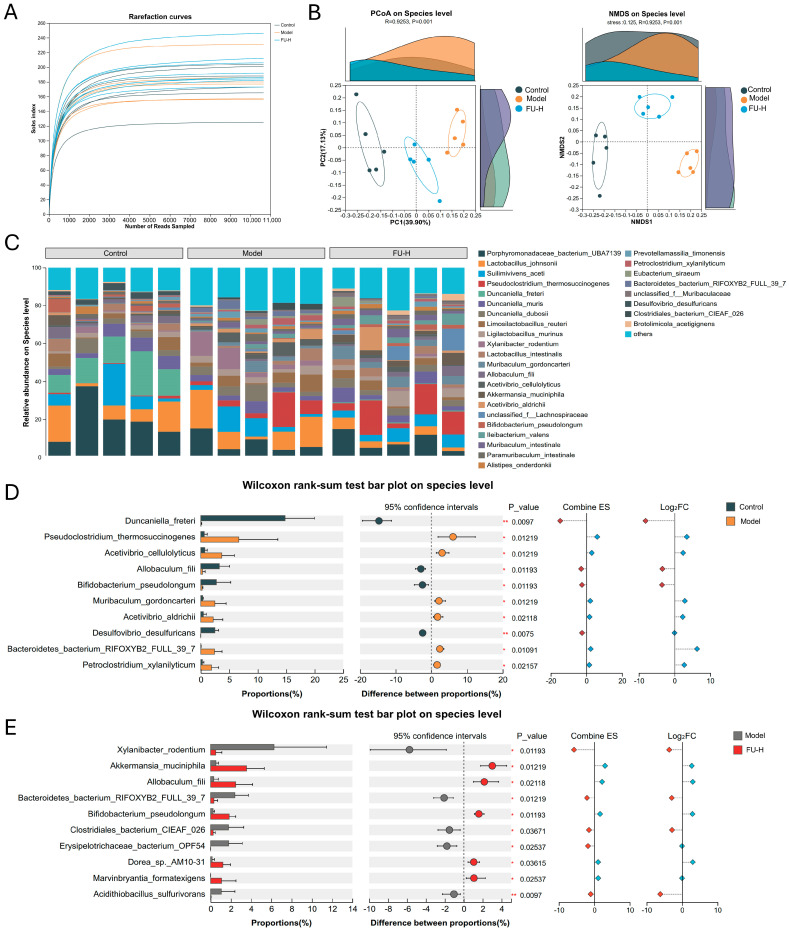
FU modulates Pb-induced gut microbiota dysbiosis. (**A**) Rarefaction curves of fecal microbiota samples from the Control, Model, and FU-H groups, indicating sequencing depth and species richness; (**B**) principal coordinate analysis (PCoA) and non-metric multidimensional scaling (NMDS) based on species-level fecal microbiota profiles, showing differences in microbial community structure among the Control, Model, and FU-H groups; (**C**) species-level microbial community composition in fecal samples from the Control, Model, and FU-H groups; (**D**) Wilcoxon rank-sum test showing differentially abundant bacterial species between the Control and Model groups; (**E**) Wilcoxon rank-sum test showing differentially abundant bacterial species between the Model and FU-H groups. Data were analyzed based on 16S rRNA gene sequencing of fecal samples. Differences in bacterial abundance were assessed using the Wilcoxon rank-sum test, and *p* < 0.05 was considered statistically significant. In the Wilcoxon rank-sum plots, bars represent the relative abundance of bacterial species, dots indicate differences between proportions with 95% confidence intervals, and the effect size and log_2_ fold change are shown on the right, * *p* < 0.05 and ** *p* < 0.01.

Given the critical role of gut microbiota in Pb-induced liver injury, we further investigated the alterations in fecal microbial composition following FU intervention. The rarefaction curves based on observed amplicon sequence variants (ASVs), as reflected by the sobs index, gradually approached a plateau, indicating that the sequencing depth was sufficient to capture the microbial diversity of the fecal samples and was suitable for subsequent analysis (Figure 6A). Principal coordinate analysis (PCoA) and non-metric multidimensional scaling (NMDS) based on Bray–Curtis distances showed clear separation of fecal microbial communities among the Control, Model, and FU groups (Figure 6B). This separation was further supported by permutational multivariate analysis of variance (PERMANOVA) analysis with 999 permutations, which confirmed significant differences in microbial community structure among the three groups (F = 5.14696, R^2^ = 0.37715, *p* = 0.001) (Table S2).

Community composition analysis illustrated the distribution of bacterial taxa assigned at the species level across groups (Figure 6C). To identify differentially abundant taxa, we performed pairwise Wilcoxon rank-sum tests with FDR correction for taxa assigned at the species level. Considering the limited species-level resolution of 16S rRNA sequencing, these taxa were interpreted cautiously as putative species-level assignments. Compared with the Control group, the Model group exhibited decreased relative abundances of taxa assigned as *Duncaniella freteri*, *Allobaculum fili*, *Bifidobacterium pseudolongum*, and *Desulfovibrio desulfuricans*, whereas the relative abundances of taxa assigned as *Pseudoclostridium thermosuccinogenes*, *Acetivibrio cellulolyticus*, *Muribaculum gordoncarteri*, and *Petroclostridium xylanilyticum* were significantly increased (Figure 6D). Notably, compared with the Model group, FU intervention significantly increased the relative abundances of taxa assigned as *Akkermansia muciniphila*, *Allobaculum fili*, *Bifidobacterium pseudolongum*, and *Marvinbryantia formatexigens*, while decreasing the relative abundances of taxa assigned as *Xylanibacter rodentium* and *Acidithiobacillus sulfurivorans* (Figure 6E).

Linear discriminant analysis effect size (LEfSe) analysis further revealed that the genus *Acetivibrio* was significantly enriched in the Model group, whereas *Pseudoclostridium* was identified as a discriminative taxon in the FU-treated group (Figure S1A). Redundancy analysis (RDA) further demonstrated that fecal microbiota in the Model group were positively correlated with liver injury markers, including ALT and AST, and pro-inflammatory cytokines, including IL-6 and TNF-α, whereas those in the FU group showed negative correlations with these parameters (Figure S1B). Finally, PICRUSt2 functional prediction analysis indicated that the differential microbiota among the three groups were mainly associated with metabolic pathways (Figure S1C).

### 2.6. FU Ameliorates Pb-Induced Metabolic Imbalance

**Figure 7:**
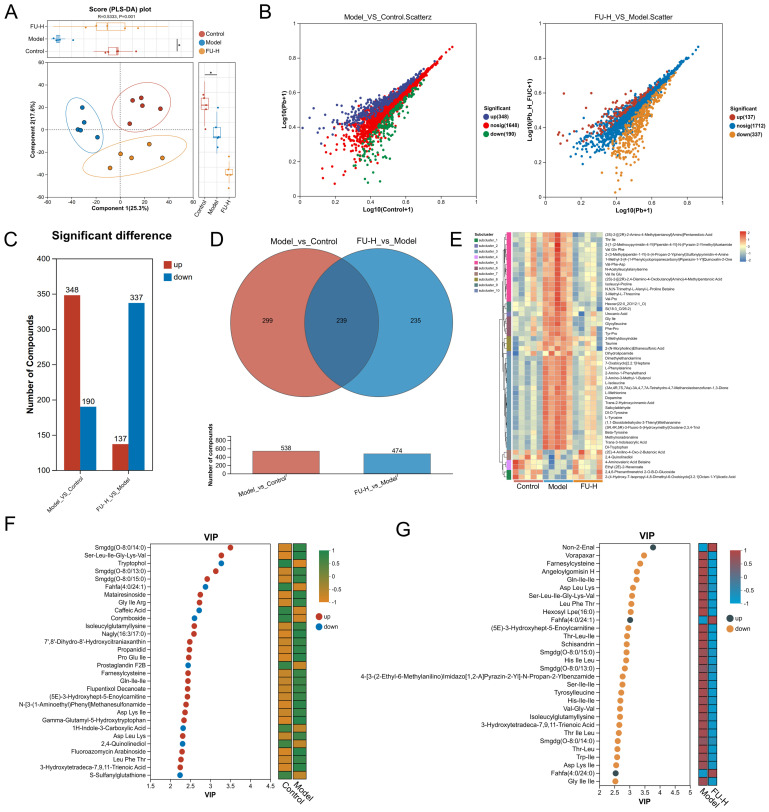
FU ameliorates Pb-induced fecal metabolic imbalance in Pb-intoxicated mice. (**A**) Partial least squares discriminant analysis (PLS-DA) score plot of fecal metabolomic profiles from the Control, Model, and FU-H groups, * *p* < 0.05; (**B**) scatter plots showing differential metabolites in the comparisons of Model versus Control and FU-H versus Model; (**C**) bar plot showing the numbers of upregulated and downregulated differential metabolites in the Model versus Control and FU-H versus Model comparisons; (**D**) Venn diagram showing the overlap of differential metabolites between the two comparisons. A total of 239 common differential metabolites were identified; (**E**) hierarchical clustering heatmap showing the expression patterns of representative differential metabolites among the Control, Model, and FU-H groups; (**F**,**G**) variable importance in projection (VIP) plots showing the top differential metabolites in the Model versus Control (**F**) and FU-H versus Model (**G**) comparisons.

To further investigate the effect of FU on the fecal metabolome in mice with Pb-induced liver injury, we performed untargeted LC-MS/MS-based metabolomic profiling. The Partial least squares discriminant analysis (PLS-DA) score plot based on the combined positive- and negative-ion-mode dataset showed distinct separation among the Control, Model, and FU-H groups, indicating marked differences in fecal metabolic profiles (Figure 7A). The reliability of the PLS-DA model was further evaluated by cross-validation and permutation testing. The model parameters showed good explanatory and predictive performance (R^2^Y = 0.996, Q^2^ = 0.878), and permutation testing indicated no obvious overfitting (*p* < 0.05) (Figure S2).

Using variable importance in projection (VIP) > 1 and FDR-adjusted *p* values < 0.05 as screening criteria, 538 putative differential metabolites were identified between the Control and Model groups, of which 348 were up-regulated and 190 were down-regulated. Between the Model and FU-H groups, 474 putative differential metabolites were identified, including 137 up-regulated and 337 down-regulated metabolites (Figure 7B,C). Venn diagram analysis showed 239 overlapping differential metabolites between these two comparisons (Figure 7D).

To visualize the importance and expression trends of the differential metabolites, VIP analysis and statistical analysis were performed on the top 30 differential metabolites using cluster heatmaps and VIP bar plots (Figure 7E–G). Compared with the Control group, the Model group exhibited decreased relative abundances of putatively annotated metabolites such as Fahfa (4:0/24:1), tryptophol, caffeic acid, and corymboside, whereas increased relative abundances of Smgdg (O-8:0/15:0), Smgdg (O-8:0/13:0), and Gin-Ile-Ile were observed. Notably, compared with the Model group, FU-H treatment increased the relative abundance of Fahfa (4:0/24:1) and decreased the relative abundances of Smgdg (O-8:0/15:0), Smgdg (O-8:0/13:0), and Gin-Ile-Ile.

**Figure 8:**
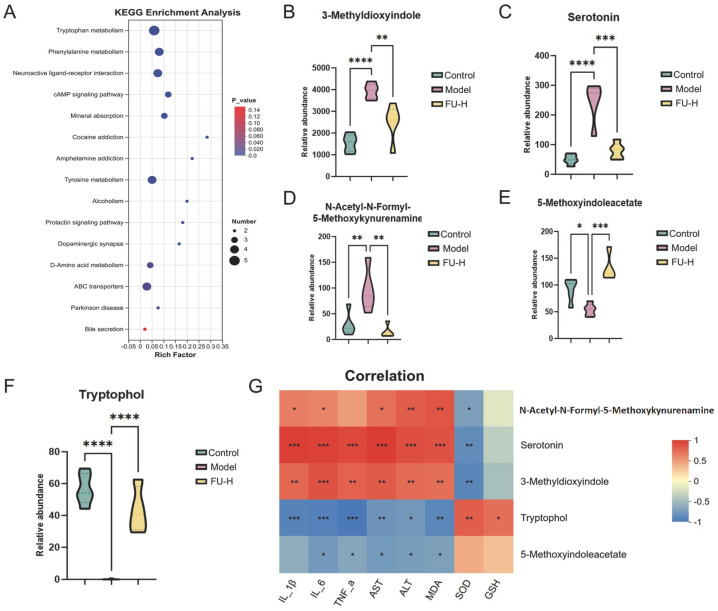
FU regulates tryptophan metabolism-related fecal metabolites associated with Pb-induced liver injury. (**A**) KEGG pathway enrichment analysis of differential fecal metabolites, showing the top 15 enriched pathways. Tryptophan metabolism was one of the prominently enriched metabolic pathways following FU intervention. Bubble size represents the number of enriched metabolites, and bubble color represents the *p* value; (**B**–**F**) relative abundance of representative tryptophan metabolism-related metabolites among the Control, Model, and FU-H groups, including 3-Methyldioxyindole (**B**), serotonin (**C**), N-Acetyl-N-Formyl-5-Methoxykynurenamine (**D**), 5-Methoxyindoleacetate (**E**), and tryptophol (**F**); (**G**) Spearman correlation heatmap showing associations between tryptophan metabolism-related metabolites and liver injury, inflammatory, and oxidative stress indicators, including AST, ALT, IL-1β, IL-6, TNF-α, MDA, SOD, and GSH. Differential metabolites were screened based on VIP > 1 and *p* < 0.05. Data are presented as violin plots or heatmaps as indicated. Statistical significance was determined by appropriate multiple-group comparison tests. Compared with the Model group: * *p* < 0.05, ** *p* < 0.01, *** *p* < 0.001, and **** *p* < 0.0001.

Subsequent KEGG pathway enrichment analysis of differential metabolites revealed that FU intervention altered multiple fecal metabolic pathways in mice with Pb-induced liver injury, among which tryptophan metabolism was the most prominent pathway (Figure 8A). Further analysis of metabolites involved in the tryptophan metabolism pathway showed that FU-H treatment increased the relative abundances of tryptophol (TOL) and 5-methoxyindoleacetate, while decreasing the relative abundances of N-acetyl-N-formyl-5-methoxykynurenamine, serotonin, and 3-methyldioxyindole (Figure 8B–F). Spearman correlation analysis further showed that, among the differential tryptophan metabolites, TOL was correlated with inflammation-related markers, including IL-6, IL-1β, and TNF-α, liver injury markers, including ALT and AST, and oxidative stress-related markers, including SOD activity, GSH level, and MDA level (Figure 8G). Taken together, these results suggest that TOL may be an important FU-associated fecal metabolite related to the alleviation of Pb-induced liver injury.

### 2.7. Exogenous Supplementation of the Tryptophan Metabolite TOL Partially Alleviates Pb-Induced Liver Injury

**Figure 9:**
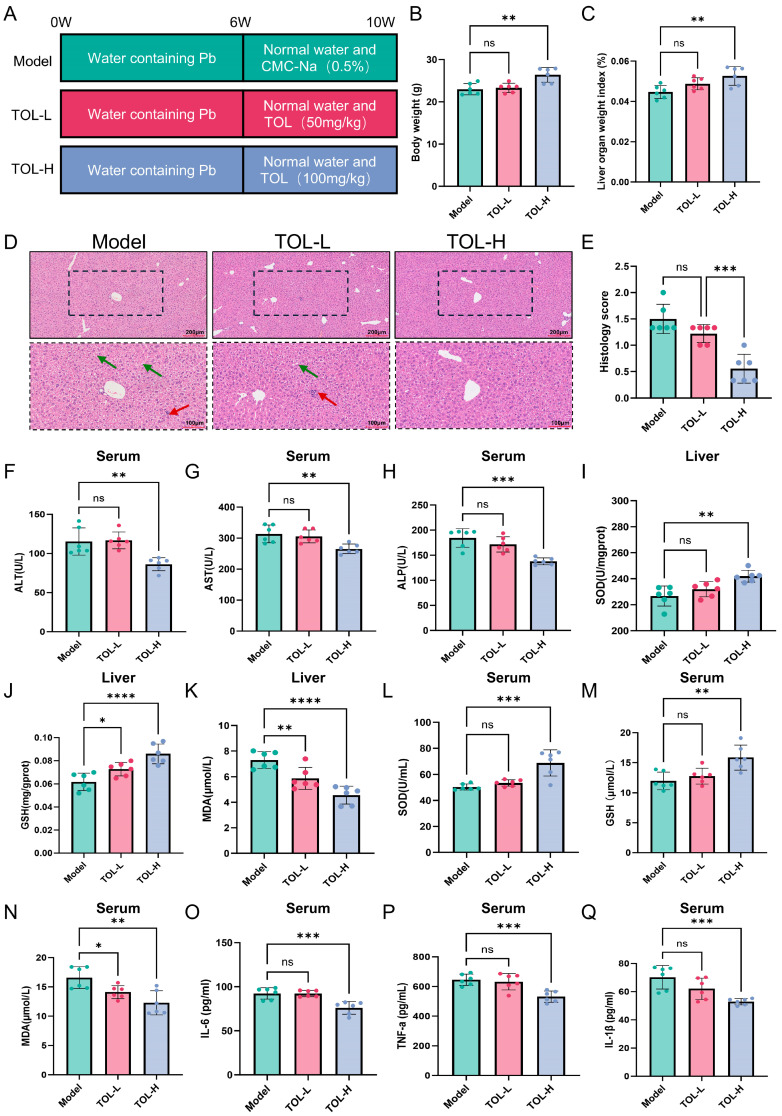
Exogenous supplementation of the tryptophan metabolite TOL partially alleviates Pb-induced liver injury. (**A**) Schematic illustration of the experimental design for TOL intervention. Pb-exposed mice were treated with vehicle, low-dose TOL (TOL-L, 50 mg/kg), or high-dose TOL (TOL-H, 100 mg/kg) for 4 weeks after 6 weeks of Pb exposure; (**B**) body weight of mice at the end of the experiment; (**C**) liver organ weight index; (**D**) representative H&E-stained liver sections from the Model, TOL-L, and TOL-H groups. The upper panels show 100×-magnification images (scale bar = 200 μm), and the lower panels show enlarged views of the dashed areas (scale bar = 100 μm). Red arrows indicate inflammatory cell infiltration, and green arrows indicate fatty vacuolation; (**E**) histology scores of liver sections; (**F**–**H**) serum levels of hepatic injury biomarkers, including ALT (**F**), AST (**G**), and ALP (**H**); (**I**–**K**) hepatic oxidative stress-related indicators, including SOD activity (**I**), GSH level (**J**), and MDA level (**K**); (**L**–**N**) serum oxidative stress-related indicators, including SOD activity (**L**), GSH level (**M**), and MDA level (**N**); (**O**–**Q**) serum levels of pro-inflammatory cytokines, including IL-6 (**O**), TNF-α (**P**), and IL-1β (**Q**). Data are presented as mean ± SD, *n* = 6 per group. Statistical significance was determined by one-way ANOVA. Compared with the Model group: ns, not significant; * *p* < 0.05, ** *p* < 0.01, *** *p* < 0.001, and **** *p* < 0.0001.

Based on the above metabolomic and correlation analyses, we further supplemented exogenous TOL to evaluate whether this tryptophan-derived metabolite may contribute to the alleviation of Pb-induced liver injury. As shown in Figure 9A, mice were treated with TOL for 4 weeks following Pb exposure. Compared with the Model group, TOL intervention increased body weight and liver index in mice with Pb-induced liver injury (Figure 9B,C). H&E staining showed that TOL supplementation alleviated Pb-induced hepatic histopathological alterations relative to the Model group (Figure 9D,E).

For serum liver injury markers, TOL-L slightly increased ALT by 1.2% but reduced AST and ALP by 2.6% and 6.9%, respectively, whereas TOL-H reduced ALT, AST, and ALP by 25.3%, 15.4%, and 25.2%, respectively, compared with the Model group (Figure 9F–H). For hepatic oxidative stress-related indicators, TOL-L restored hepatic SOD and GSH by 2.3% and 17.8%, respectively, and decreased hepatic MDA by 19.6%, while TOL-H restored hepatic SOD and GSH by 6.7% and 39.1%, respectively, and decreased hepatic MDA by 37.5% (Figure 9I–K). Similar changes were observed in serum oxidative stress-related indicators, where TOL-L restored serum SOD and GSH by 6.2% and 6.7%, respectively, and decreased serum MDA by 15.0%, whereas TOL-H restored serum SOD and GSH by 36.6% and 32.4%, respectively, and decreased serum MDA by 25.9% (Figure 9L–N). In addition, TOL-L reduced serum IL-6, TNF-α, and IL-1β by 0.2%, 2.0%, and 11.6%, respectively, whereas TOL-H reduced these cytokines by 17.8%, 17.6%, and 24.7%, respectively, compared with the Model group (Figure 9O–Q). These results suggest that exogenous TOL supplementation, especially at the high dose, partially alleviated Pb-induced liver injury, oxidative stress dysregulation, and systemic inflammatory responses.

## 3. Discussion

With the continuous advancement of global industrialization, Pb contamination has become an urgent global public health concern [3]. As the central organ responsible for metabolism and detoxification, the liver is highly susceptible to Pb accumulation, which may ultimately lead to hepatic dysfunction. Although chelation therapy remains the main clinical strategy for treating Pb poisoning, its application is limited by potential adverse effects and the incomplete recovery of established target-organ injury [10,33]. In this context, dietary natural products have attracted increasing attention as potential adjunctive interventions for heavy metal exposure because of their relatively low toxicity, multi-target effects, and suitability for long-term use [34,35]. In the present study, FU, a sulfated polysaccharide derived from brown algae, alleviated Pb-induced liver injury in mice. This protective effect may be associated with reduced Pb burden, improvement of oxidative stress and inflammatory responses, regulation of IGFBP1-related molecular responses, remodeling of gut microbiota, and modulation of tryptophan metabolism.

The present findings are consistent with toxic manifestations commonly associated with Pb exposure. Pb-exposed mice showed decreased body weight, reduced food intake, and obvious liver injury, which were closely related to systemic Pb accumulation [22]. FU intervention alleviated these Pb-induced toxic phenotypes. Notably, FU reduced Pb levels in serum and liver tissues while increasing fecal Pb content, suggesting that FU may alter Pb distribution in exposed mice and may be related to intestinal Pb elimination. However, fecal Pb content can be affected by food intake, fecal mass, gastrointestinal transit, and sample collection timing. Therefore, the current data cannot directly demonstrate that FU promotes Pb excretion.

Serum ALT, AST, and ALP are well-recognized biomarkers for evaluating liver injury. A previous study confirmed that lead acetate intoxication significantly elevates serum ALT and AST activities [22]. Consistent with these findings, Pb exposure increased serum ALT, AST, and ALP levels in the present study, whereas FU intervention reduced these liver injury markers. H&E staining further showed disordered hepatocyte arrangement, inflammatory cell infiltration, and extensive fatty vacuolation in the Model group, which were consistent with the histopathological alterations reported by Abd Elrasoul et al. in Pb-exposed animals [36]. FU intervention attenuated these pathological changes, indicating that FU can alleviate Pb-induced hepatic functional and histological injury under the present experimental conditions.

Oxidative stress is a core mechanism underlying lead acetate-triggered hepatotoxicity [37]. In the present study, Pb exposure decreased SOD activity and GSH levels, while increasing MDA accumulation in serum and liver tissue, suggesting the occurrence of systemic and hepatic oxidative stress-related alterations. FU intervention partially reversed these changes, indicating that FU may improve redox homeostasis in Pb-exposed mice. Superoxide anions and hydroxyl radicals can cause severe damage to cellular and tissue structures [38]; therefore, enhancement of antioxidant defense is an important strategy for alleviating Pb-induced liver injury. Previous studies have shown that the sulfate groups and fucose residues of FU may contribute to ROS scavenging through electron- or hydrogen-donating mechanisms. In addition, FU has been reported to regulate the expression of antioxidant enzyme-related genes, including SOD, glutathione peroxidase (GPx), and catalase (CAT), thereby improving antioxidant capacity [39]. FU can also suppress lipid peroxidation product accumulation and protect cell membranes and organelles from oxidative damage [40]. These findings are consistent with previous studies showing that FU protects against liver injury by improving liver function, reducing hepatocyte steatosis and inflammatory infiltration, and enhancing antioxidant status [41,42].

Integrated liver transcriptome sequencing and network toxicology analysis suggested that the MAPK signaling pathway may be involved in FU-associated hepatoprotection. Previous studies have reported that excessive activation of the MAPK/ERK pathway can promote ROS accumulation and aggravate oxidative damage [43]. Among the candidate genes related to this pathway, IGFBP1 showed marked expression changes after FU intervention. Notably, previous studies have also highlighted the regulatory role of MAPK/ERK signaling in the transcriptional regulation of downstream IGFBP1 expression [32]. IGFBP1 is a liver-enriched secreted protein that mainly regulates the bioavailability of IGF-1 [15]. Dysregulated IGFBP-1 expression has been documented in multiple liver diseases, including alcoholic liver disease [16] and NAFLD, and is closely correlated with increased senescent cell accumulation in NAFLD models [17]. In addition, the role of IGFBP1 in liver injury appears to be context-dependent. For example, in a carbon tetrachloride-induced liver injury model, IGFBP1 knockout aggravated liver injury and increased hepatocyte death, suggesting that IGFBP1 participates in hepatocyte survival and liver repair [18]. Conversely, another study reported that taurine alleviated carbon tetrachloride-induced liver injury by inhibiting IGFBP1 expression and reducing lipid peroxidation and MDA levels [19]. In the present study, RT-qPCR validated the expression changes of representative candidate genes and MAPK pathway-related genes. Moreover, exogenous administration of recombinant IGFBP1 protein partially weakened the protective effects of FU on oxidative stress-related indicators and liver injury phenotypes, suggesting that IGFBP1 is involved in FU-associated hepatoprotection.

Mounting studies have confirmed that gut microbiota is essential for maintaining liver homeostasis, while Pb exposure severely disrupts microbial equilibrium [40,44]. The present study demonstrated that FU alleviates Pb-induced liver injury by regulating gut microbiota and tryptophan metabolism via the gut–liver axis. The abundance of beneficial bacteria, such as *Bifidobacterium pseudolongum*, was significantly decreased in the model group, whereas FU intervention restored the abundance of *Akkermansia muciniphila* and *Bifidobacterium pseudolongum*. Redundancy analysis further showed that the gut microbiota composition of Pb-exposed mice was positively correlated with liver injury markers and pro-inflammatory cytokines, whereas the FU-associated microbial profile was negatively correlated with these pathological indicators. These results suggest that gut microbiota remodeling is closely associated with the hepatoprotective effects of FU.

The biological functions of gut microbiota depend largely on their metabolic profiles [45]. Untargeted metabolomics analysis revealed that FU intervention profoundly altered fecal metabolic pathways in Pb-exposed mice, with the tryptophan metabolic pathway showing the most prominent changes. Abnormal tryptophan metabolism has been widely reported in NAFLD patients and animal models, and differential tryptophan metabolites (e.g., indolelactic acid, xanthurenic acid) are closely associated with gut dysbiosis and disease severity [46]. Microbial tryptophan metabolites can activate the AhR/Nrf2 pathway, thereby alleviating hepatic oxidative stress and liver sinusoidal endothelial cell injury [47], which confirms the regulatory role of tryptophan metabolism in hepatic oxidative homeostasis. Further Spearman correlation analysis demonstrated that among all differential tryptophan metabolites, only TOL was significantly correlated with liver injury, inflammation, and oxidative stress markers. TOL belongs to indole derivatives, which is consistent with previous reports that gut microbiota metabolizes tryptophan into indole compounds to regulate host physiological functions [48]. Wikoff et al. also demonstrated that circulating indole metabolites under physiological conditions are predominantly derived from microbial metabolism [49]. In our in vivo experiments, exogenous TOL supplementation dose-dependently improved body weight and liver index, alleviated hepatic pathological lesions, reduced serum ALT, AST, ALP and inflammatory cytokine levels, and restored SOD activity while decreasing MDA content in Pb-exposed mice. These results suggest that TOL may be an important FU-associated fecal metabolite related to the alleviation of Pb-induced liver injury.

Overall, by integrating multi-omics approaches, this study provides preliminary evidence for the potential protective mechanisms of FU against Pb-induced liver injury from multiple perspectives, including Pb burden, hepatic oxidative stress, IGFBP1-related molecular responses, gut microbiota remodeling, and tryptophan metabolism. These findings provide new experimental evidence for the use of natural polysaccharides as potential adjunctive interventions for heavy metal exposure-related liver injury. However, several limitations should be acknowledged. First, although sulfate, hydroxyl, and carboxyl-related groups in FU may confer potential metal-binding capacity, and FU decreased Pb levels in serum and liver while increasing fecal Pb content in the present study, the formation of FU–Pb complexes was not directly verified. In addition, classical chelating agents such as DMSA or CaNa_2_EDTA were not included as positive controls. Therefore, the Pb-lowering effect of FU cannot be directly compared with clinically used Pb chelation therapies, and the precise mechanism by which FU reduces Pb burden remains to be further clarified. Future studies should combine chelator controls and in vitro Pb-binding assays to distinguish the potential direct Pb-binding effect of FU from its indirect hepatoprotective effects related to oxidative stress, inflammation, and gut microbiota-derived metabolism.

Second, although the Pb-exposure model used in this study reproduced key features of Pb-induced liver injury and has been widely applied in studies of Pb hepatotoxicity [34], it cannot fully mimic the long-term, low-dose chronic environmental exposure that is more common in human Pb poisoning. Notably, only male mice were used in the present study, and potential sex-related differences in Pb-induced hepatotoxicity and FU responsiveness were not evaluated. In addition, because a FU-only group was not included, the systemic safety of long-term FU administration under non-Pb-exposed conditions remains to be further assessed. Therefore, future studies should establish chronic low-dose Pb exposure models in both male and female animals and include FU-only groups to determine whether the hepatoprotective effects of FU are stable under long-term exposure conditions and to further evaluate its long-term safety.

Finally, the mechanistic conclusions of this study require further causal validation. Although the present results support the involvement of IGFBP1 and TOL in FU-associated hepatoprotection, liver-specific IGFBP1 knockout or intervention models, fecal microbiota transplantation, antibiotic-treated models, or germ-free animal models are still needed to verify their causal roles. Moreover, although previous studies have provided basic characterization data for the commercial FU preparation used in this study, including its main-chain structure, infrared spectrum, compositional features, and sulfate content, FU has obvious structural heterogeneity. Its biological activity may be jointly determined by multiple structural features, including sulfation degree, fucose-rich backbone, molecular weight, branching pattern, and monosaccharide composition. Future studies using FU fractions with different sulfation degrees, molecular weights, and monosaccharide compositions, as well as chemically modified derivatives such as desulfated FU, are needed to further clarify the structure–activity relationship underlying its hepatoprotective effects.

## 4. Materials and Methods

### 4.1. Chemicals and Reagents

Fucoidan used in this study was a commercially available high-purity preparation purchased from MedChemExpress (MCE; Monmouth Junction, NJ, USA; HY-132179; purity, 98.62%). The product information and purity data were obtained from the certificate of analysis provided by the supplier. Kang et al. reported that the main chain of this fucoidan reagent consists of repeating α-L-fucopyranose residues linked by (1 → 4) glycosidic bonds, and also provided Fourier-transform infrared spectroscopy (FTIR) characterization, compositional analysis, and sulfate content data for this preparation [50]. Sodium carboxymethyl cellulose (CMC-Na, HY-Y1889A), recombinant mouse IGFBP1 protein (HY-P700256), and tryptophol (TOL, HY-W010155) were also purchased from MedChemExpress (MCE; Monmouth Junction, NJ, USA). Lead acetate [Pb(C_2_H_3_O_2_)_2_] was obtained from Sigma-Aldrich (St. Louis, MO, USA). All reagents were prepared and stored strictly according to the manufacturers’ instructions. Commercial enzyme-linked immunosorbent assay (ELISA) kits for mouse IL-6, TNF-α, and IL-1β were purchased from MultiSciences Biotech Co., Ltd. (Hangzhou, Zhejiang, China). Assay kits for SOD, MDA, and GSH were supplied by Suzhou Comin Biotechnology Co., Ltd. (Suzhou, Jiangsu, China). A mouse IGFBP1 ELISA kit (ab272465) was obtained from Abcam (Cambridge, UK). TRIzol reagent, the reverse transcription kit, and SYBR Green-based quantitative PCR reagents used for RT-qPCR validation were purchased from Invitrogen (Carlsbad, CA, USA).

### 4.2. Experimental Design and Sample Collection

Male specific pathogen-free (SPF) C57BL/6 mice aged 5 weeks were purchased from Beijing Vital River Laboratory Animal Technology Co., Ltd. (Beijing, China). All mice were acclimatized for 1 week before the experiments under controlled environmental conditions, including a temperature of 22 ± 1 °C, relative humidity of 40–60%, and a 12 h light/dark cycle. During the acclimatization period, mice had free access to standard chow and drinking water. All animal procedures were approved by the Institutional Animal Care and Use Committee of The First Hospital of Jiaxing (Affiliated Hospital of Jiaxing University) (Approval No. JXYY2026-097) and were performed in accordance with the National Institutes of Health Guide for the Care and Use of Laboratory Animals.

For each independent animal experiment, mice were randomly assigned to the indicated experimental groups using a computer-generated random number table after the acclimatization period. Body weights were balanced among groups before treatment to minimize baseline differences. During sample collection and subsequent biochemical, histological, and omics analyses, samples were labeled with coded identifiers. Investigators responsible for histological evaluation, biochemical measurements, and data analysis were blinded to the group allocation until the primary analyses were completed. Because the treatment solutions and administration procedures differed among some intervention groups, complete blinding during gavage and injection was not feasible. However, outcome assessment was performed in a blinded manner.

#### 4.2.1. Experiment 1: Protective Effect of FU Against Pb-Induced Liver Injury

Mice were randomly divided into 4 groups (*n* = 6 per group): Control, Model, FU-L, and FU-H. Mice in the Control group received a normal diet and regular drinking water throughout the experiment. Mice in the Model, FU-L, and FU-H groups received the same diet but were provided with drinking water containing 1.5 g/L lead acetate for 6 weeks to establish the Pb-exposure model [34]. Thereafter, the lead-containing water was replaced with normal drinking water. Mice in the FU-L and FU-H groups were orally gavaged with FU at doses of 100 and 200 mg/kg body weight (b.w.), respectively [51], suspended in 0.2 mL of 0.5% CMC-Na, once daily for 4 consecutive weeks. Mice in the Model group received an equivalent volume of 0.5% CMC-Na. Food intake was recorded weekly during the experiment. At 2 h after the final gavage, mice were weighed and euthanized. The liver was excised and weighed, and the liver organ index was calculated as the liver-to-body weight ratio according to the following formula: liver organ index (%) = liver weight (g)/body weight (g) × 100. Liver tissues were fixed in 4% buffered formalin for histological analysis, and additional liver samples were collected for hepatic lead quantification, hepatic oxidative stress marker assessment, RNA sequencing, and reverse transcription quantitative PCR validation. Peripheral blood was collected by cardiac puncture for serum biochemical analysis and determination of inflammatory cytokines, serum oxidative stress-related indicators, and lead content. Fecal samples were collected for 16S rRNA gene sequencing, untargeted metabolomic analysis, and fecal lead measurement.

#### 4.2.2. Experiment 2: Role of IGFBP1 in FU-Mediated Protection Against Pb-Induced Liver Injury

A separate cohort of male SPF C57BL/6 mice was randomly assigned to 3 groups (*n* = 6 per group): Model, FU-H, and IGFBP1+FU-H. All mice were provided with drinking water containing 1.5 g/L lead acetate for 6 weeks and then switched to normal drinking water, as described in Experiment 1. Subsequently, mice in the FU-H and IGFBP1+FU-H groups received daily oral gavage of FU at 200 mg/kg b.w., suspended in 0.2 mL of 0.5% CMC-Na, for 4 weeks. Mice in the Model group received an equal volume of 0.5% CMC-Na. During the same intervention period, mice in the IGFBP1+FU-H group received recombinant mouse IGFBP1 protein at a dose of 2 μg per mouse in 50 μL sterile saline via tail vein injection every 4 days, whereas mice in the FU-H group received an equal volume of sterile saline via the same route. At the end of the experiment, body weight and liver organ index were recorded. Blood samples were collected by cardiac puncture for serum biochemical analysis and determination of inflammatory cytokines, serum oxidative stress-related indicators, and IGFBP1 levels. Liver tissues were collected for histological analysis and hepatic oxidative stress marker assessment as described above.

#### 4.2.3. Experiment 3: Protective Effect of Tryptophol Against Pb-Induced Liver Injury

To evaluate the protective effect of TOL against Pb-induced liver injury, mice were randomly divided into 3 groups (*n* = 6 per group): Model, TOL-L, and TOL-H. The Pb-induced liver injury model was established as described in Experiment 1 by administering drinking water containing 1.5 g/L lead acetate for 6 weeks. After Pb exposure, mice were switched to normal drinking water. Mice in the TOL-L and TOL-H groups were orally gavaged with TOL at doses of 50 and 100 mg/kg b.w. [52], respectively, suspended in 0.2 mL of 0.5% CMC-Na, once daily for 4 weeks. Mice in the Model group received 0.2 mL of 0.5% CMC-Na alone. At the end of the experiment, body weight and liver organ index were recorded. Blood samples were collected for serum biochemical analysis, inflammatory cytokine measurement, and serum oxidative stress-related indicator assessment. Liver samples were collected for histological examination and hepatic oxidative stress marker assessment.

### 4.3. Determination of Lead Content

Pb content in blood, liver, and fecal samples was determined as previously described [22], with minor modifications. Briefly, blood, liver tissue, and fecal samples collected at the end of the experiment were aliquoted for analysis. Liver tissue and fecal samples were accurately weighed before digestion. Each sample was digested with 1 mL of nitric acid and 0.5 mL of hydrofluoric acid, followed by gradual heating at 100 °C until complete digestion was achieved. After cooling to room temperature, the digested solution was diluted to a fixed volume with ultrapure water. The Pb concentration in the digested samples was then measured using inductively coupled plasma mass spectrometry (ICP-MS; Agilent, USA) according to the manufacturer’s standard operating procedures.

To ensure the reliability of Pb quantification, external calibration was performed using Pb standard solutions covering the expected concentration range of the digested samples, and the calibration curve showed good linearity with a coefficient of determination (R^2^) greater than 0.999. Procedural blanks and reagent blanks were included to monitor potential background contamination. Quality-control samples were inserted into the analytical sequence, and selected samples were measured in duplicate to assess analytical precision. The limits of detection and quantification were determined according to the instrument response and blank measurements. Spike-recovery tests were performed to evaluate method accuracy, and the recovery and relative standard deviation values were within generally accepted ranges for ICP-MS-based Pb determination.

### 4.4. Histological Analysis of the Liver

Liver tissues were freshly harvested and fixed in 4% paraformaldehyde for 24 h. After fixation, the tissues were embedded in paraffin and cut into 4-μm-thick sections. The sections were stained with hematoxylin and eosin (H&E) for histopathological evaluation. Images were captured using an Olympus light microscope, and scale bars were added to the representative images.

Liver histological scoring was performed based on lipid accumulation and inflammatory changes using Image-Pro Plus software (version 6.0, Media Cybernetics, Rockville, MD, USA), as previously applied in other studies [22]. Steatosis was assessed by quantifying the lipid droplet area with Image-Pro Plus, and the percentage of liver parenchyma occupied by medium and large intracytoplasmic lipid droplets was scored as follows: <5% = 0, 5–33% = 1, 34–66% = 2, and >66% = 3. Lobular inflammation was scored according to the number of inflammatory foci: none = 0, <2 foci = 1, 2–4 foci = 2, and >4 foci = 3. Hepatocyte ballooning was scored as follows: none = 0, few ballooned cells = 1, and many ballooned cells = 2. Histological scoring was performed in a blinded manner.

### 4.5. Biochemical Analysis

At the end of the experiment, whole blood was collected and centrifuged at 1000× *g* for 15 min to separate serum. Serum levels of ALT, AST, and ALP were measured using an automatic biochemical analyzer (BS-200, Mindray, Shenzhen, China) to evaluate liver function impairment.

### 4.6. Measurement of Oxidative Stress in Serum and Liver Tissue

Oxidative stress-related indicators in serum and liver tissue were assessed using commercial assay kits. For serum samples, whole blood was collected in sterile anticoagulant-free tubes and centrifuged at 1000× *g* for 10 min to separate serum. For liver tissue samples, liver tissues were homogenized in ice-cold buffer solution and then centrifuged at 4 °C and 8000× *g* for 10 min. The supernatant was collected for subsequent analysis. The activity of SOD and the levels of GSH and MDA in both serum and liver tissue supernatants were measured according to the manufacturers’ protocols.

### 4.7. Measurement of Inflammatory Factors

Serum levels of inflammatory cytokines were measured using commercial ELISA kits. Briefly, whole blood was allowed to clot at room temperature for 30 min and then centrifuged at 1000× *g* for 10 min. The supernatant serum was collected, and the concentrations of IL-6, TNF-α, and IL-1β were determined according to the manufacturer’s instructions.

### 4.8. Determination of IGFBP1 Levels in Peripheral Blood

Peripheral blood was collected from each mouse via cardiac puncture. Blood samples were allowed to clot at room temperature for 30 min and then centrifuged at 1000× *g* for 15 min to obtain serum. Serum IGFBP1 levels were measured using a commercial mouse IGFBP1 ELISA kit following the manufacturer’s instructions.

### 4.9. Network Toxicology Analysis

To elucidate the molecular targets associated with Pb-induced liver injury, a network toxicology approach was performed. Both Pb-related targets and liver injury-related targets were collected from the CTD and GC databases. The overlapping targets between Pb exposure and liver injury were identified as the candidate targets of Pb-induced hepatotoxicity. Hub genes were screened based on degree centrality. GO functional enrichment and Kyoto Encyclopedia of KEGG pathway enrichment analyses were performed using the clusterProfiler package in R (version 4.0), with a significance threshold of *p* < 0.05.

### 4.10. RNA Sequencing

After the experiment, fresh liver tissues were collected from each group and immediately stored at −80 °C until RNA extraction. Total RNA was extracted using TRIzol reagent (Invitrogen, USA) according to the manufacturer’s instructions. RNA concentration and purity were assessed using a NanoDrop spectrophotometer, and RNA integrity was evaluated using an Agilent 2100 Bioanalyzer (Agilent, Santa Clara, CA, USA). Only RNA samples with acceptable purity and integrity were used for library construction. Library preparation and RNA sequencing were performed by Shanghai Majorbio Bio-pharm Biotechnology Co., Ltd. (Shanghai, China). Raw sequencing reads were quality-filtered using fastp (v1.1.0), and clean reads were aligned to the mouse reference genome using HISAT2. Gene expression levels were quantified and normalized using RSEM. Differential expression analysis was performed using the DESeq2 package in R. DEGs were screened using the criteria of |log_2_ fold change| ≥ 1 and Benjamini–Hochberg adjusted *p* value < 0.05. GO functional enrichment and KEGG pathway enrichment analyses of DEGs were performed using the clusterProfiler package in R, with an adjusted *p* value < 0.05 considered statistically significant.

### 4.11. Reverse Transcription Quantitative PCR Validation

To validate the RNA sequencing and network toxicology results, RT-qPCR was performed to examine the mRNA expression levels of representative candidate genes and MAPK signaling pathway-related genes. Total RNA was extracted from liver tissues using TRIzol reagent according to the manufacturer’s instructions. RNA concentration and purity were determined using a NanoDrop spectrophotometer. The extracted RNA was then reverse-transcribed into complementary DNA (cDNA) using a reverse transcription kit following the manufacturer’s protocol. RT-qPCR was performed using SYBR Green-based quantitative PCR reagents on a real-time PCR detection system. The reaction conditions were set according to the manufacturer’s instructions. Relative mRNA expression levels were calculated using the 2−ΔΔCt method, with Gapdh used as the internal reference gene. The primer sequences used for RT-qPCR are listed in Table S1.

### 4.12. 16S rRNA Sequencing and Gut Microbiota Analysis

To further investigate the alterations in fecal microbial community composition following fucoidan intervention in mice with Pb-induced liver injury, fecal samples were collected from each group at the end of the experiment. Fecal microbial genomic DNA was extracted using a commercial DNA extraction kit according to the manufacturer’s instructions. The V3–V4 hypervariable region of the bacterial 16S rRNA gene was amplified using specific primers. Library construction and sequencing were performed by Shanghai Majorbio Bio-pharm Biotechnology Co., Ltd. (Shanghai, China) on an Illumina MiSeq platform (Illumina, San Diego, CA, USA).

Raw sequencing reads were quality-filtered and trimmed using fastp (v1.1.0), and high-quality reads were processed using QIIME2 (v2022.2). After denoising, chimera removal, and quality control, ASVs were generated using the DADA2 algorithm. Taxonomic annotation was performed against the SILVA database (v138.2). Before diversity analysis, all samples were rarefied to an even sequencing depth. Rarefaction curves and Good’s coverage values indicated that the sequencing depth was sufficient for subsequent microbiota analysis.

β diversity was calculated based on Bray–Curtis distances and visualized using PCoA and NMDS. Differences in microbial community structure among groups were assessed using PERMANOVA with 999 permutations. Pairwise comparisons of taxa assigned at the species level were performed using the Wilcoxon rank-sum test. *p* values were adjusted for multiple comparisons using the Benjamini–Hochberg false discovery rate (BH-FDR) method, and taxa with FDR-adjusted *p* values < 0.05 were considered differentially abundant. LEfSe was used to identify discriminative taxa among groups, with a Kruskal–Wallis test *p* value < 0.05 and an LDA score threshold > 2. Microbial functional profiles were predicted using PICRUSt2 (v2.6.2). Considering the limited species-level resolution of 16S rRNA sequencing, taxa annotated at the species level in this study were cautiously interpreted as putative species-level assignments.

### 4.13. Untargeted Metabolomics Analysis and Data Processing

To investigate fecal metabolite alterations in mice with Pb-induced liver injury following fucoidan intervention, fecal samples were collected from each group at the end of the experiment. Untargeted metabolomics analysis was performed by Shanghai Majorbio Bio-pharm Biotechnology Co., Ltd. (Shanghai, China) using LC-MS/MS. Briefly, fecal metabolites were extracted using an appropriate solvent system. The extracted samples were then separated by ultra-performance liquid chromatography and analyzed using a high-resolution mass spectrometer.

Raw LC-MS/MS data were processed on the Majorbio Cloud Platform for peak detection, peak alignment, data normalization, and metabolite annotation. Metabolite annotation was performed by matching accurate molecular mass, isotope distribution patterns, and MS/MS fragmentation information against public metabolomics databases, including HMDB, KEGG, and the Majorbio in-house database. In the absence of validation using authentic standards, all annotated metabolites were considered putatively identified metabolites according to metabolomics identification confidence criteria.

PLS-DA was performed to visualize metabolic profile differences among groups. The reliability of the PLS-DA models was evaluated by 200 permutation tests combined with cross-validation. The model parameters R^2^Y and Q^2^ were used to assess goodness of fit and predictive ability, respectively. Differential metabolites were screened using VIP > 1 and BH-FDR-adjusted *p* < 0.05. Metabolic pathway enrichment analysis was performed based on the KEGG database.

### 4.14. Statistical Analysis

Statistical analyses were performed using GraphPad Prism 8.0.1 (GraphPad Software, New York, NY, USA). Data are presented as the mean ± standard deviation (SD). The number of biological replicates is indicated in the corresponding figure legends. For normally distributed data with homogeneous variances, comparisons between two groups were performed using an unpaired two-tailed Student’s *t*-test, whereas comparisons among multiple groups were performed using one-way analysis of variance (ANOVA) followed by Tukey’s multiple-comparison test. For repeated-measures data, such as food intake, two-way ANOVA was used. Correlation analyses were performed using Spearman’s correlation analysis.

For descriptive reporting of treatment effects, percentage inhibition or recovery was calculated based on group mean values. For indicators increased by Pb exposure, including ALT, AST, ALP, MDA, and inflammatory cytokines, percentage inhibition was calculated as follows: (Model group mean − treatment group mean)/Model group mean × 100%. For indicators decreased by Pb exposure, including SOD and GSH, percentage recovery was calculated as follows: (treatment group mean − Model group mean)/Model group mean × 100%. For the IGFBP1 intervention experiment, the percentage reversal was calculated relative to the FU-H group mean according to the direction of change of each indicator.

For omics datasets, multiple-testing correction was performed as described in the corresponding RNA sequencing, 16S rRNA sequencing, and metabolomics methods sections. *p* values or adjusted *p* values < 0.05, as appropriate, were considered statistically significant.

## 5. Conclusions

**Figure 10:**
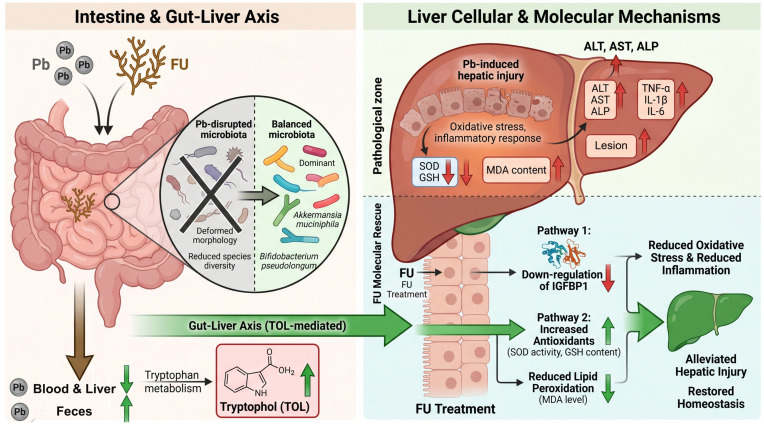
Schematic illustration of the potential mechanisms associated with FU-mediated attenuation of Pb-induced liver injury. Pb exposure disrupts gut microbiota homeostasis, induces microbial dysbiosis, and increases systemic Pb burden, thereby contributing to hepatic oxidative stress, inflammatory responses, and liver injury. FU treatment decreased Pb levels in serum and liver and increased fecal Pb content. In the intestine, FU treatment was associated with remodeling of Pb-disrupted gut microbiota, including increased relative abundance of beneficial bacteria such as Akkermansia muciniphila and Bifidobacterium pseudolongum, as well as alterations in fecal tryptophan metabolism, particularly tryptophol (TOL). Through the gut–liver axis, these FU-associated intestinal changes may contribute to the alleviation of Pb-induced hepatic injury. In the liver, FU protection was associated with IGFBP1-related redox modulation, increased antioxidant capacity, including SOD activity and GSH content, reduced lipid peroxidation, as reflected by decreased MDA levels and suppressed inflammatory responses, including TNF-α, IL-1β, and IL-6. These coordinated changes were accompanied by reduced serum ALT, AST, and ALP levels, alleviated hepatic pathological lesions, and improved hepatic homeostasis.

In conclusion, the present study showed that FU alleviated Pb exposure-induced liver injury in mice and reduced Pb accumulation, liver dysfunction, inflammatory responses, and oxidative stress-related alterations. The protective effects of FU may be associated with reduced Pb burden, regulation of IGFBP1-related molecular responses, remodeling of gut microbiota structure, and modulation of the gut microbiota-derived tryptophan metabolite TOL (Figure 10). These findings provide experimental evidence supporting FU as a potential dietary adjunctive intervention for Pb exposure-related liver injury and offer a reference for further elucidating the mechanisms by which natural polysaccharides regulate heavy metal-induced hepatotoxicity.

## Data Availability

All data are available in the main text or the Supplementary Materials.

## Supplementary Materials

The following supporting information can be downloaded at: https://www.mdpi.com/article/10.3390/md24070232/s1, Figure S1: Gut microbiota profiling and functional prediction analyses in Pb-induced mice and FU intervention; Figure S2: Validation of the PLS-DA/OPLS-DA model by permutation testing; Table S1: Primer sequences used for quantitative real-time PCR; Table S2: PERMANOVA analysis.

## Abbreviations

The following abbreviations are used in this manuscript:

FU	Fucoidan
Pb	Lead
ROS	Reactive Oxygen Species
IGFBP1	Insulin-Like Growth Factor-Binding Protein 1
TOL	Tryptophol
ALT	Alanine Aminotransferase
AST	Aspartate Aminotransferase
ALP	Alkaline Phosphatase
SOD	Superoxide Dismutase
GSH	Glutathione
MDA	Malondialdehyde
IL-6	Interleukin-6
TNF-α	Tumor Necrosis Factor-α
IL-1β	Interleukin-1β
RNA-Seq	RNA Sequencing
DEGs	Differentially Expressed Genes
GO	Gene Ontology
KEGG	Kyoto Encyclopedia of Genes and Genomes
CTD	Comparative Toxicogenomics Database
AhR	Aryl Hydrocarbon Receptor
GPx	Glutathione Peroxidase
NAFLD	Non-Alcoholic Fatty Liver Disease
ELISA	Enzyme-Linked Immunosorbent Assay
SPF	Specific Pathogen-Free
SD	Standard Deviation
GC	GeneCards Database
MAPK	Mitogen-Activated Protein Kinase
PI3K	Phosphatidylinositol 3-Kinase
Akt	Protein Kinase B
PCoA	Principal Coordinate Analysis
NMDS	Non-Metric Multidimensional Scaling
NCM	Neutral Community Model
LEfSe	Linear Discriminant Analysis Effect Size
RDA	Redundancy Analysis
PCA	Principal Component Analysis
PLS-DA	Partial Least Squares Discriminant Analysis
OPLS-DA	Orthogonal Partial Least Squares Discriminant Analysis
VIP	Variable Importance in Projection
LC-MS/MS	Liquid Chromatography–Tandem Mass Spectrometry
UPLC	Ultra-Performance Liquid Chromatography
OTUs	Operational Taxonomic Units
ASVs	Amplicon Sequence Variants
FXR	Farnesoid X Receptor
TGR5	Takeda G Protein-Coupled Receptor 5
Nrf2	Nuclear Factor Erythroid 2-Related Factor 2
CAT	Catalase
H&E	Hematoxylin and Eosin
ICP-MS	Inductively Coupled Plasma Mass Spectrometry
CMC-Na	Sodium Carboxymethyl Cellulose
