# Supplementary material for: Fucoidan Attenuates Lead-Induced Liver Injury Associated with IGFBP1 and Gut Microbiota-Derived Tryptophol Metabolism
Source: Mar Drugs. 2026 Jul 2;24(7):232. doi: 10.3390/md24070232 (PMC13413114; doi:10.3390/md24070232)
Supplement: Supplementary file 1 [file marinedrugs-24-00232-s001.zip › Table S1.pdf]

TableS1: Primer sequences used for quantitative real-time PCR

Gene	Primer Sequence (5'→3')
Igfbp1	F: ATCAGCCCATCCTGTGGAAC<br>R: TGCAGCTAATCTCTCTAGCACTT
Ppp1r10	F: AGCTGGACACCCCATCTTTG<br>R: GGACAGGGGCTGAATTGAGA
Dnajb9	F: CTCCACAGTCAGTTTTCGTCTT<br>R: GGCCTTTTTGATTTGTCGCTC
Hspa1a	F: TGGTGCAGTCCGACATGAAG<br>R: GCTGAGAGTCGTTGAAGTAGGC
Cyp2a4	F: TCACCATCTATCTGGGATCTCG<br>R: CCCCGAAGACGATTGAGCTAA
Cbx2	F: GGCTGGTCCTCCAAACACAA<br>R: CCCTGGGTCTCTTGCCTCT
Gadd45a	F: CCGAAAGGATGGACACGGTG<br>R: TTATCGGGGTCTACGTTGAGC
Egr1	F: TCGGCTCCTTTCCTCACTCA<br>R: CTCATAGGGTTGTTCGCTCGG
Map2k1	F: AAGGTGGGGGAACTGAAGGAT<br>R: CGGATTGCGGGTTTGATCTC
Map2k2	F: GTTACCGGCACTCACTATCAAC<br>R: CCTCCAGCCGCTTCCTTTG
Mapk1	F: TGACCCTTATGACCAGTCCTTT<br>R: GTCAGGCTCTTCCACTCATCTAT
Mapk3	F: TCCGCCATGAGAATGTTATAGG<br>R: GGTGGTGTTGATAAGCAGATTGG
Gapdh	F: AGGTCGGTGTGAACGGATTTG<br>R: TGTAGACCATGTAGTTGAGGTCA
