# Supplementary material for: Fucoidan Attenuates Lead-Induced Liver Injury Associated with IGFBP1 and Gut Microbiota-Derived Tryptophol Metabolism
Source: Mar Drugs. 2026 Jul 2;24(7):232. doi: 10.3390/md24070232 (PMC13413114; doi:10.3390/md24070232)
Supplement: Supplementary file 1 [file marinedrugs-24-00232-s001.zip › Table S2.pdf]

Name	SumsOfSqs	MeanSqs	F.Models	R2	Pr(>F)
Control_Model_FU_H	1.0152	0.5076	5.14696	0.37715	0.001
