# Supplementary figures and images for: Fucoidan Attenuates Lead-Induced Liver Injury Associated with IGFBP1 and Gut Microbiota-Derived Tryptophol Metabolism
Source: Mar Drugs. 2026 Jul 2;24(7):232. doi: 10.3390/md24070232 (PMC13413114; doi:10.3390/md24070232)

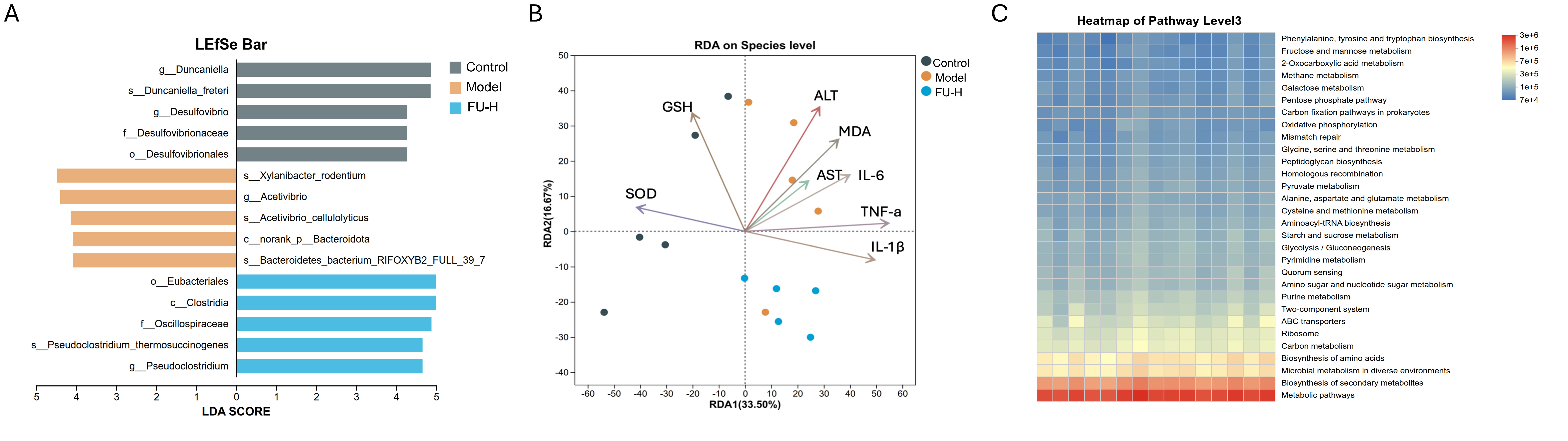

Supplement: Supplementary file 1 [file marinedrugs-24-00232-s001.zip › Figure S1.jpg]

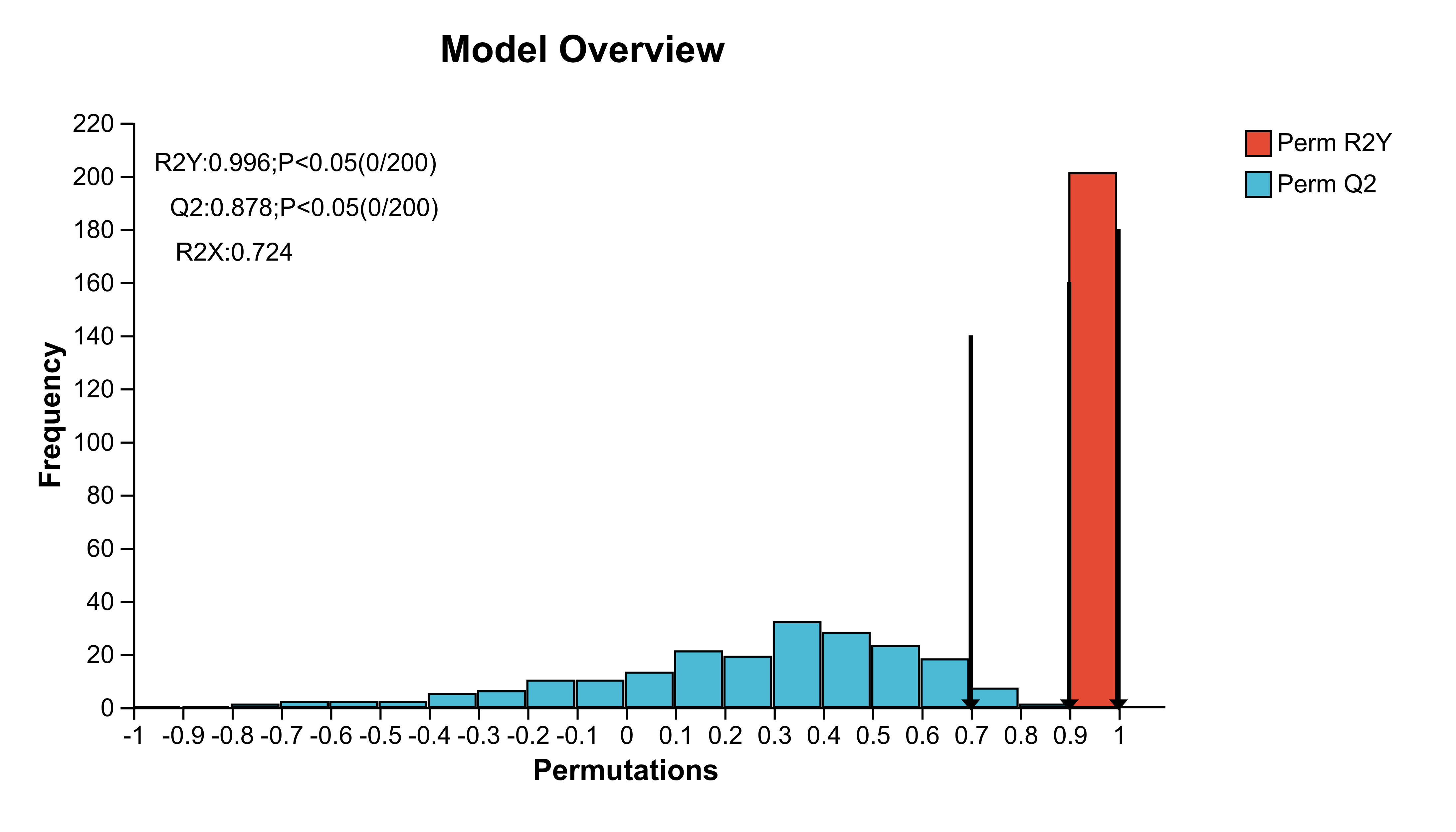

Supplement: Supplementary file 1 [file marinedrugs-24-00232-s001.zip › Figure S2.png]
